# Complement component 3 (C3) expression in the hippocampus after excitotoxic injury: role of C/EBPβ

**DOI:** 10.1186/s12974-016-0742-0

**Published:** 2016-10-21

**Authors:** Elena Hernandez-Encinas, Diana Aguilar-Morante, Jose A. Morales-Garcia, Elena Gine, Marina Sanz-SanCristobal, Angel Santos, Ana Perez-Castillo

**Affiliations:** 1 Instituto de Investigaciones Biomédicas, (CSIC-UAM), Arturo Duperier, 4, 28029 Madrid, Spain; 2 Centro de Investigación Biomédica en Red sobre Enfermedades Neurodegenerativas (CIBERNED), 28031 Madrid, Spain; 3 Departamento de Bioquímica y Biologia Molecular, Facultad de Medicina, UCM, 28040 Madrid, Spain; 4 Departamento de Biología Celular, Facultad de Medicina, UCM, 28040 Madrid, Spain; 5 Present Address: Departamento de Fisiología Médica y Biofísica, Instituto de Biomedicina de Sevilla, IBiS, (Hospital Universitario Virgen del Rocío/CSIC/Universidad de Sevilla), 41013 Sevilla, Spain

**Keywords:** C/EBPβ, C3, Excitotoxicity, Neurodegeneration, Neuroinflammation

## Abstract

**Background:**

The CCAAT/enhancer-binding protein β (C/EBPβ) is a transcription factor implicated in the control of proliferation, differentiation, and inflammatory processes mainly in adipose tissue and liver; although more recent results have revealed an important role for this transcription factor in the brain. Previous studies from our laboratory indicated that CCAAT/enhancer-binding protein β is implicated in inflammatory process and brain injury, since mice lacking this gene were less susceptible to kainic acid-induced injury. More recently, we have shown that the complement component 3 gene (C3) is a downstream target of CCAAT/enhancer-binding protein β and it could be a mediator of the proinflammatory effects of this transcription factor in neural cells.

**Methods:**

Adult male Wistar rats (8–12 weeks old) were used throughout the study. C/EBPβ^+/+^ and C/EBPβ^–/–^ mice were generated from heterozygous breeding pairs. Animals were injected or not with kainic acid, brains removed, and brain slices containing the hippocampus analyzed for the expression of both CCAAT/enhancer-binding protein β and C3.

**Results:**

In the present work, we have further extended these studies and show that CCAAT/enhancer-binding protein β and C3 co-express in the CA1 and CA3 regions of the hippocampus after an excitotoxic injury. Studies using CCAAT/enhancer-binding protein β knockout mice demonstrate a marked reduction in C3 expression after kainic acid injection in these animals, suggesting that indeed this protein is regulated by C/EBPβ in the hippocampus in vivo.

**Conclusions:**

Altogether these results suggest that CCAAT/enhancer-binding protein β could regulate brain disorders, in which excitotoxic and inflammatory processes are involved, at least in part through the direct regulation of C3.

**Electronic supplementary material:**

The online version of this article (doi:10.1186/s12974-016-0742-0) contains supplementary material, which is available to authorized users.

## Background

CCAAT/enhancer-binding protein β (C/EBPβ) is a member of a family of transcription factors whose members contain a basic leucine-zipper domain for DNA binding and dimerization [1, 2]. C/EBPβ exists in three isoforms generated from a single messenger RNA (mRNA) due to alternative translation initiation sites [3, 4]. C/EBPβ is expressed in numerous tissues, including liver, adipose tissue, kidney, lung, ovary, mammary gland, and hematopoietic tissues, and regulates a variety of biological processes, including metabolism, proliferation and differentiation (depending on the cell context), and immune response [1, 5–8].

In the nervous system, C/EBPβ is essential for regulating numerous processes such as synaptic plasticity and long-term memory [9, 10], neuronal differentiation [11, 12], neuroinflammation and excitotoxicity [13–16], and hippocampal neurogenesis [17]. Also, due to its relevance in the indicated cellular processes, C/EBPβ is also involved in the pathogenesis of different diseases, e.g., cancer, hyper-inflammatory processes, and bacterial infections [1, 18, 19]. As it happens in normal physiological conditions, this regulation takes place via the regulation of many genes involved in these processes [15–17, 20, 21].

The complement pathway is an essential regulator of the immune response, including chemotaxis, phagocytosis, cell adhesion, and B and T cell differentiation [22, 23], and it is a very important line of defense against infections through the elimination of invading pathogens and regulation of the adaptive immune response [24, 25]. Regarding the central nervous system, several studies have shown that the complement system plays also important roles in the central nervous system that extend far beyond host defense and inflammatory processes [26–32]. Specifically, complement component 3 (C3), a central component of the complement cascade, is a critical mediator of synaptic refinement and plasticity [31, 33] and localizes at synapsis where it mediates its pruning during development [31, 34], normal aging [35], and neurodegeneration [36, 37]. In fact, mice deficient in C3 exhibit deficits in synaptic remodeling, increased synaptic connectivity, and enhanced epileptiform activity due to failed synaptic pruning [38]. It has been also shown that C3 regulates hippocampal neurogenesis in adult mammalian brain [39] and C3-deficient mice present impaired neurogenesis following cerebral ischemia [40, 41].

Besides its role in normal physiological conditions, also uncontrolled complement activation in the brain has been associated with various neurodegenerative disorders, including Alzheimer’s disease, Parkinson’s disease, Huntington’ disease, dementia, and multiple sclerosis [30, 42–50]. Increased levels of C3 have been found in the cerebrospinal fluid (CSF) of patients with Parkinson’s and Alzheimer’s diseases, and these levels augment with the progression of the disease [49]. Zanjani et al. showed that C3 localized surrounding β-amyloid plaques in early stages of Alzheimer’s disease, when an important loss of synapsis takes place [51], and other authors have demonstrated a modulatory role of C3 on amyloid pathology in animal models of Alzheimer’s disease [52, 53]. However, little is known about the mechanisms regulating C3 expression and its influence on neuronal function and dysfunction in the adult brain.

Taking into account the previous results from our laboratory showing a direct regulation of C3 by C/EBPβ in neural cells [54], here we examined the possible regulation by C/EBPβ of C3 expression in the hippocampus in an animal model of excitotoxicity. For this purpose, we have analyzed the expression of both C/EBPβ and C3 after an excitotoxic injury induced by kainic acid (KA) injection in the hippocampus of rodents (rats and mice). Our results reveal a strong induction of C/EBPβ and C3 in the CA1 and CA3 regions of the hippocampus following kainic acid injection. We also show a pronounced reduction of C3 levels in *C/EBPβ* knockout mice, which have reduced neurotoxicity [16]. These data point to a role for C3 as a possible mediator of the effects of C/EBPβ in inflammatory and excitotoxic processes in vivo.

## Methods

### Animals

Adult male Wistar rats (8–12 weeks old) were used throughout the study. C/EBPβ^+/+^ and C/EBPβ^–/–^ mice were generated from heterozygous breeding pairs, kindly provided by C. M. Croniger and R. W. Hanson (Case Western Reserve University, Cleveland, OH) [55]. Genotypes were identified using genomic PCR, with DNA prepared from tail using the REDExtract-N-AmpTM tissue PCR kit (XNAT kit, Sigma, St Louis, MO). Five animals from each experimental group were analyzed. All procedures with animals were specifically approved by the “Ethics Committee for Animal Experimentation” of the Instituto de Investigaciones Biomédicas and carried out in accordance with the European Communities Council, directive 2010/63/EEC and National regulations, normative 53/2013. Special care was taken to minimize pain or discomfort of animals.

### Kainic acid injection in vivo

Adult male rats, C/EBPβ^+/+^ and C/EBPβ^–/–^ mice, were anesthetized by intraperitoneal injection of ketamine (60 mg/kg) and medetomidine (0.125 mg/kg) and positioned in a stereotaxic apparatus (Kopf Instruments, CA). Kainic acid (KA) (0.25 μg in 2.50 μl PBS) was delivered unilaterally into the left hippocampus. Flow rate (1 μl/min) was kept constant with a motorized syringe pump (BASi), and the needle was kept in place for 2 min post-injection before being slowly withdrawn. Control animals of the same age were injected with vehicle. Animals were then housed individually to recover and sacrificed 72 h after lesioning with KA.

### Quantitative real-time-PCR

Total rat or mouse hippocampal RNA samples (2 μg) were used for the synthesis of complementary DNA (cDNA) by reverse transcription using the Reverse Transcription System (Promega, Madison, WI, USA) with a pd(N)6 random hexamer. Real-time PCR was performed in an ABI Prism machine using the SYBR Green PCR Master Mix (Applied Biosystems, Warrington, UK) and 300 nM concentrations of specific primer. The primers used for the determination of the concentration of both rat and mouse C3 mRNA were 5′-ACC TTA CCT CGG CAA GTT TCT-3′(forward sequence) and 5′-TTG TAG AGC TGC TGG TCA GG-3′(reverse sequence), which synthesized a fragment of 140 bp. In all runs, melting curves were performed to ensure that only one DNA fragment was amplified. Cycle threshold (dilution 1:10) was around 25. Amplification of the 18S rRNA was used for normalization of cDNA loading in the PCR as previously described [56]. The relative mRNA content was determined with the 2-ΔΔCt method [57].

### Immunohistochemistry

Animals previously anesthetized were perfused transcardially with 4 % paraformaldehyde, and the brains were removed, postfixed, and processed for immunohistochemistry using the diaminobenzidine (DAB) method or double-immunofluorescence analysis, as previously described [58]. The following primary antibodies were used: rabbit polyclonal anti-C3 (Abcam, Cambridge, UK), mouse monoclonal anti-C/EBPβ (Abcam, Cambridge, UK), rabbit polyclonal anti-NeuN (Merck-Millipore, Darmstadt, Alemania), rabbit polyclonal anti-IL-1β (Abcam), rat monoclonal anti-CD11b (clon OX42, Serotec, Oxford, UK), and rabbit polyclonal anti-glial fibrillary acidic protein (GFAP, Dako, Glostrup, Denmark) for immunodetection of astrocytes. For immunofluorescence analysis to detect C3, a secondary Alexa-Fluor488 goat anti-rabbit was used in combination with Texas Red Lycopersicon esculentum (tomato lectin; Vector Labs USA, emission at 546) to label microglial cells or Neurotrace fluorescent Nissl stain (emission at 546; Molecular Probes, Madrid, Spain) to stain neurons. The slides processed with DAB were examined with a Nikon Eclipse 80i (Düsseldorf, Germany) microscope, equipped with a Nikon DS-Fi1 digital camera. For double immunofluorescence, a LSM710 laser scanning spectral confocal microscope (Zeiss) was used. Confocal microscope settings were adjusted to produce the optimum signal-to-noise ratio. Orthogonal image acquisition was performed as previously described [59]. Briefly, sections containing the CA3 region of the hippocampus were used for the analysis. Confocal acquisitions with orthogonal projections show co-localization of C3/neurotrace, C3/GFAP, and C3/tomato lectin, over the extent of the cell in consecutive 0.5- or 2-μm z-stacks.

A differential interference contrast (DIC) microscope was used to identify the morphology of cells. The images were acquired using a Nikon 90i microscope equipped with a Plan Apo 100× Ph3 DM objective and connected to Nis-Elements BR software.

### Fluoro-Jade staining

To evaluate neuronal degeneration, Fluoro-Jade B staining was used [60]. Briefly, sections mounted in gelatin-coated slides were immersed in 100 % alcohol, followed by 70 % alcohol and distilled water containing permanganate. Slides were then incubated with 0.001 Fluoro-Jade B dye (Chemicon, Temecula, USA) for 30 min at room temperature. After staining, sections were washed with distilled water and mounted with DePeX (Serva).

### TUNEL staining

Frozen hippocampal sections were mounted in gelatin-coated slides. The TUNEL protocol was performed using the In Situ Cell Death Detection Kit, POD (Roche Diagnostics, Indianapolis, USA), following the manufacturer’s instructions. Slides were mounted with Vectashield (Vector Laboratories), and TUNEL staining was visualized and imaged using a Nikon Eclipse 80i (Düsseldorf, Germany) microscope, equipped with a Nikon DS-Fi1 digital camera.

## Results

### Kainic acid induced neurodegeneration in the CA3 region of the hippocampus in rats and mice

**Fig. 1:**
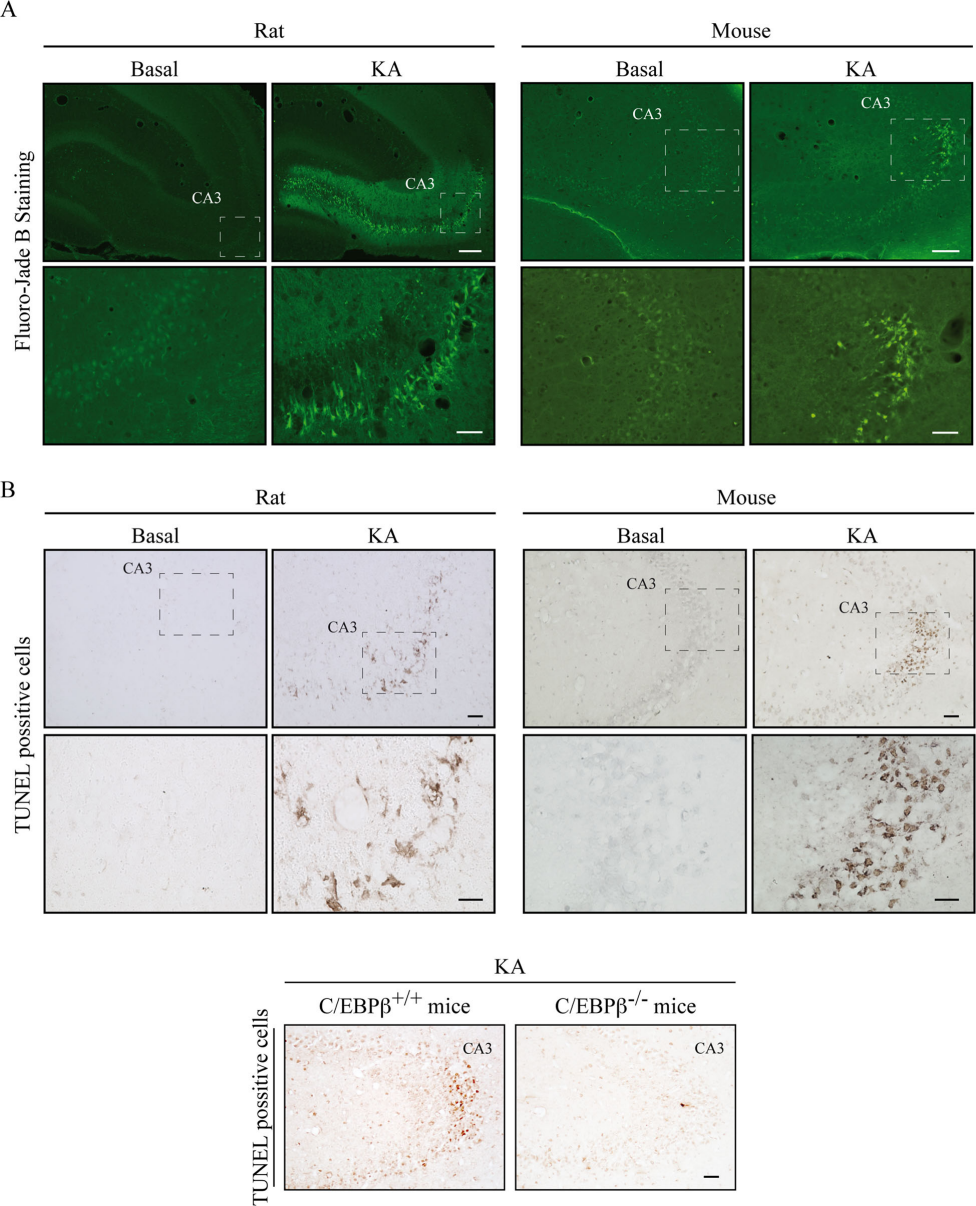
Excitotoxic brain injury induced by KA injection in the hippocampus. Animals were injected in the right hemisphere with KA and sacrificed 72 h post-injection. **a** Representative images of Fluoro-Jade B staining in the CA3 region of the hippocampus. *Scale bars*, 200 μm (rat) and 250 μm (mouse). *Inset scale bars*, 50 μm (rat) and 25 μm (mouse). **b** Representative images of TUNEL staining in the CA3 region of the hippocampus. *Scale bar*, 50 μm. *Inset scale bar*, 25 μm

First, we validated our models of excitotoxicity by analyzing the damaging effect of KA in the CA3 subfield, which is the most sensitive area of the hippocampus to this excitotoxin. As expected, we found a dramatic increase in the number of degenerating neurons stained with Fluoro-Jade B (Fig. 1a) in rats and mice injected with KA, which was more noticeable in the pyramidal cells of this area. We also found that this cell death occurs via apoptosis since a significant increase in the number of TUNEL^+^ cells (Fig. 1b) was also observed, which was not detected in C/EBPβ-deficient mice (Fig. 1b).

### KA induces the expression of C/EBPβ and C3 genes in the hippocampus of adult rats

**Fig. 2:**
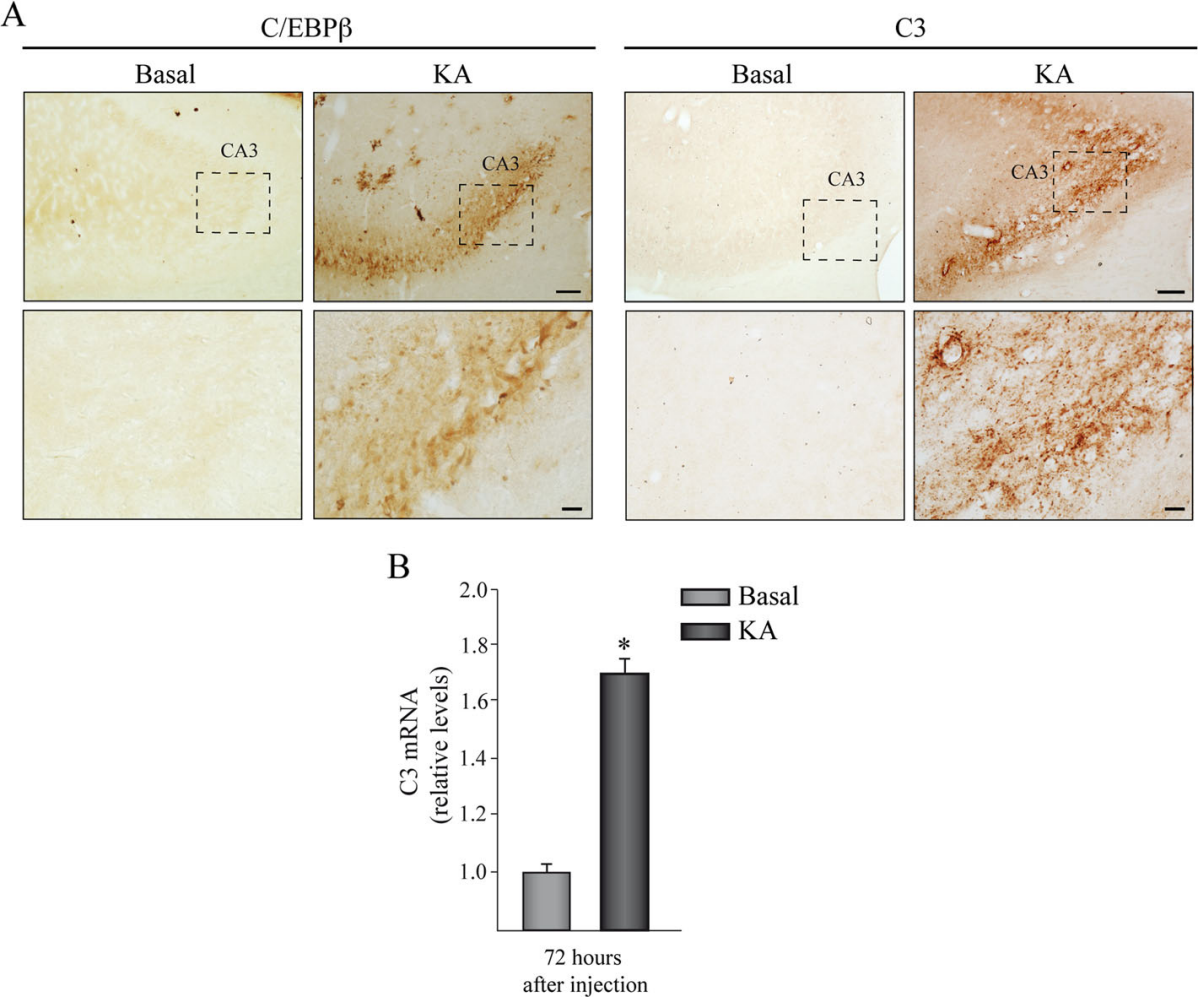
Induction of C/EBPβ and C3 expression after KA injection in adult rats. Animals were injected in the right hemisphere with KA and sacrificed 72 h post-injection. **a** Immunohistochemical analysis of coronal brain sections using specific anti-C/EBPβ and anti-C3 antibodies, showing the CA3 field of the hippocampus. *Scale bar*, 100 μm. *Inset scale bar*, 25 μm. **b** RT-PCR analysis of C3 mRNA content in the hippocampus of adult rats treated or not with KA. The graphic shows the mean ± SD of three different experiments. *P < 0.05

We next studied the content of C/EBPβ and C3 proteins, by immunohistochemistry analysis, in consecutive slices of the hippocampus of adult rats after KA injury. As shown in Fig. 2a, C/EBPβ and C3 proteins are barely detectable in the hippocampus of control animals. In contrast, after KA injection, a dramatic increase of both proteins was observed, which was most prominent in the CA3 subfield of the hippocampus (Fig. 2a). No expression of either C/EBPβ or C3 was detected in other hippocampal areas. It is known that the injection of KA can cause the rupture of the blood brain barrier (BBB) allowing the entry of plasmatic proteins into the brain. Therefore, we next analyzed whether the C3 protein found in the CA3 region after KA injection comes from outside the central nervous system or is due to a local synthesis in the hippocampus. To this end, we studied the hippocampal amount of C3 mRNA by quantitative PCR. Figure 2b shows that in fact C3 mRNA was also increased after KA injection indicating that the observed increase in C3 protein is caused, at least in part, by an increase in the production of C3 gene in brain cells.

### KA induces C3 expression in neurons and glial cells in the hippocampus of adult rats

**Fig. 3:**
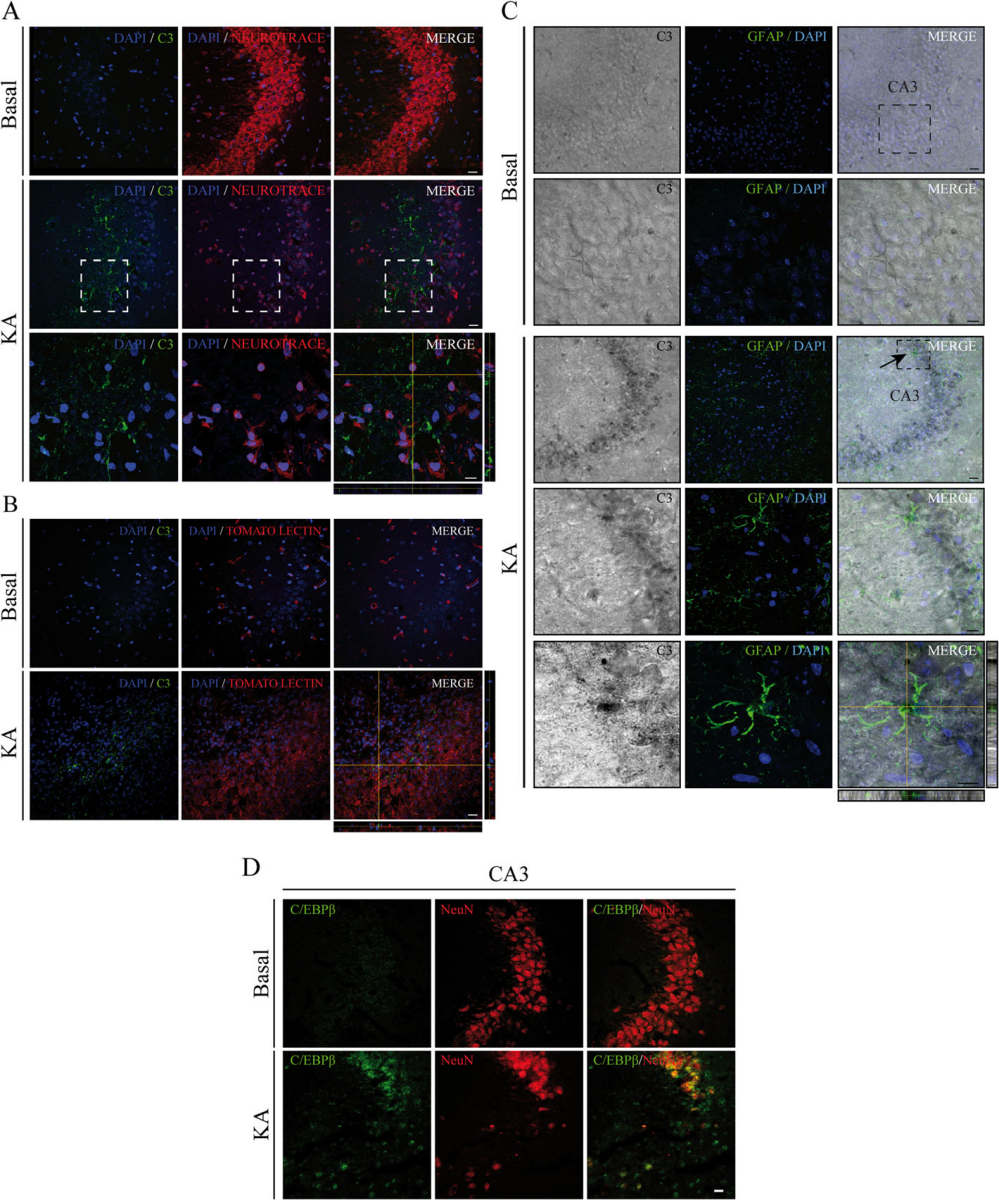
Induction of C3 expression after KA injection in neurons and glial cells in the CA3 region of the hippocampus of adult rats. **a** Double fluorescent immunohistochemistry showing C3 expression (*green*) near hippocampal neurons labeled with neurotrace (*red*). **b** Representative images showing the expression of C3 (*green*) and microglial cells stained with tomato lectin (*red*). **c** Immunohistochemistry performed using a fluorescent antibody against GFAP (*green*) together with a DAB-stained anti-C3. *Scale bar*, 20 μm. *Inset scale bar*, 10 μm. DAPI was used as a nuclear marker. All images represent the maximum intensity projection, and orthogonal views are also shown, generated by projecting *z*-series in the *x*- and *y*-planes. **d** Double-immunofluorescence images showing C/EBPβ expression after KA injection in neuronal cells (NeuN-positive cells). *Scale bar*, 50 μm

Next we analyzed the cell types responsible for the KA-induced increase in C3 observed in the rat hippocampus. For that purpose, we performed double immunostaining in the CA3 region of the rat hippocampus, the region were the induction of C3 is most prominent. As shown in Fig. 3a, double labeling with a C3-specific antibody and neurotrace, a specific marker for neurons, clearly indicates the presence of C3 protein inside the neurons and also in the surrounding area 72 h after KA injection. Given the massive neuronal cell death that takes place in this region of the hippocampus, these observations suggest that C3 may be localized in those neurons that are already degenerating.

After brain injury, there is an increase in the number of reactive glial cells, both active astrocytes and microglial cells as labeled with a GFAP-specific antibody and tomato lectin, respectively. As expected, after KA injection, we observed an increase of both types of reactive glial cells. The presence of C3 protein was detected in both microglial cells and astrocytes, as shown in Fig. 3b, c, respectively. In addition, double-immunofluorescence analysis shows that the increase in C/EBPβ levels observed in wild type animals takes place in neurons (Fig. 3d).

Interestingly an increase in C3 labeling was clearly observed surrounding the microvasculature of the KA-injected hippocampus (Additional file 1) suggesting an implication of this protein in angiogenesis. Additionally, in this Figure, after double staining with a C3-specific antibody and tomato lectin, we show images of C3 localization near microglial cells suggesting also the opsonization and phagocytosis functions of C3 and microglial cells.

### KA induces the expression of C/EBPβ and C3 genes in the hippocampus of adult mice

**Fig. 4:**
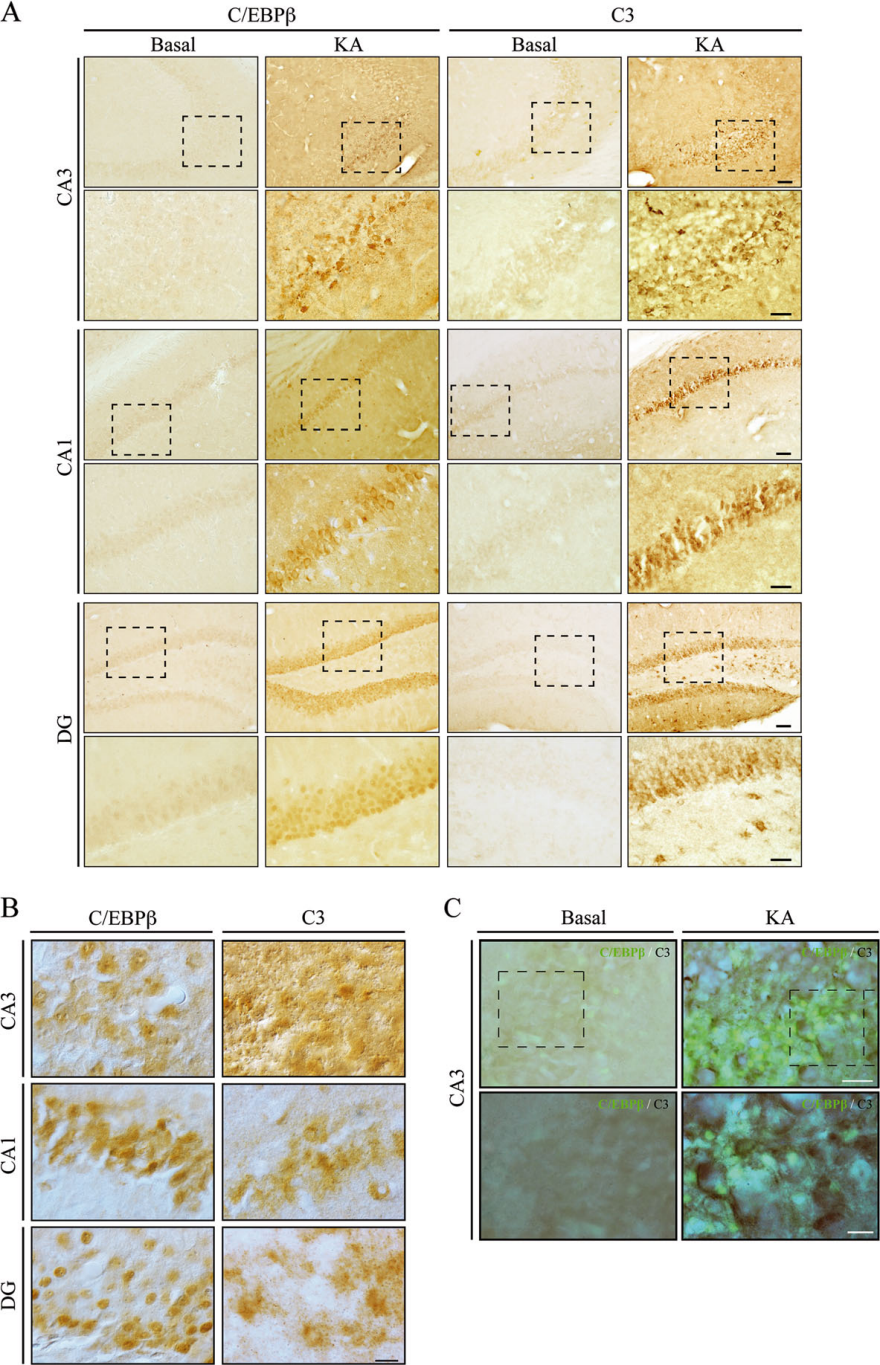
Induction of C/EBPβ and C3 expression after KA injection in adult mice. Animals were injected in the right hemisphere with KA and sacrificed 72 h post-injection. **a** Immunohistochemical analysis of coronal brain sections using specific anti-C/EBPβ and anti-C3 antibodies, showing the CA3, CA1, and DG fields of the hippocampus. *Scale bar*, 50 μm. *Inset scale bar*, 25 μm. **b** Differential interference contrast images of coronal brain sections using specific anti-C/EBPβ and anti-C3 antibodies, showing the CA3, CA1, and DG fields of the hippocampus. *Scale bar*, 10 μm. **c** Representative images showing coexpression of C3 (DAB staining) and C/EBPβ (fluorescence) in the CA3 region. *Scale bar*, 20 μm. *Inset scale bar*, 10 μm

Next, we analyzed whether the coexpression of C3 and C/EBPβ in the same areas of the hippocampus also took place in mice. We find also a dramatic increase in the amount of C/EBPβ and C3 proteins in the hippocampus after KA injection. As shown in Fig. 4a, both proteins are almost undetectable in controls in the diverse regions of the hippocampus analyzed. A clear increase is observed after KA injection; however, and in contrast with rats, we could detect C/EBPβ and C3 proteins in three different regions of the hippocampus, CA1, CA3, and dentate gyrus (DG). Figure 4b shows differential interference contrast (DIC) images of C/EBPβ and C3 to show the whole cell. A combination of DAB (C3) and fluorescence (C/EBPβ) staining shows that the increase of both proteins occurs in the same cells (Fig. 4c).

### The absence of C/EBPβ blocks the induction of C3 by KA in the mouse hippocampus

**Fig. 5:**
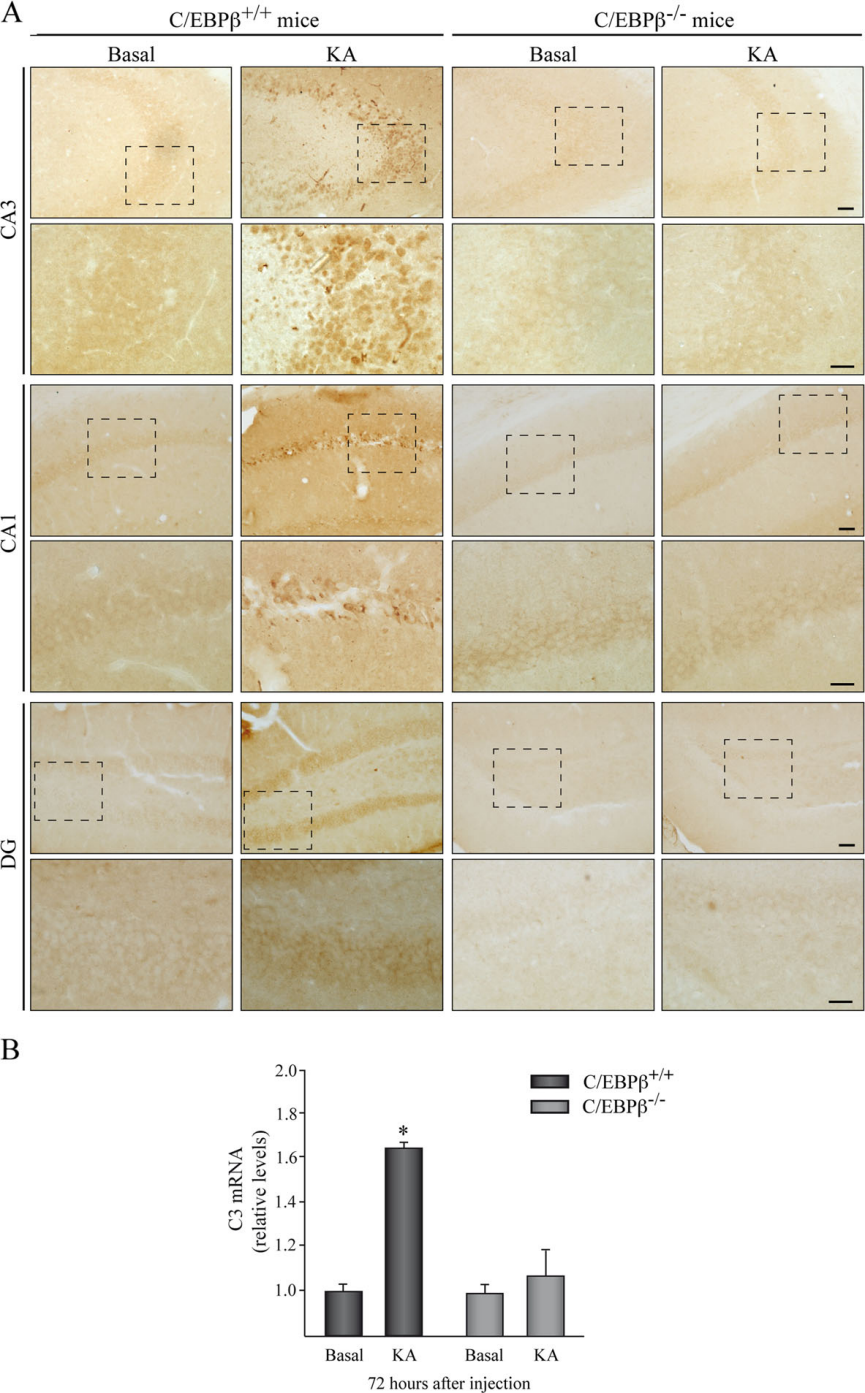
Expression of C3 in C/EBPβ wild type and knockout mice. Mice were injected with KA in the right hemisphere, sacrificed 72 h later. **a** Immunohistochemistry analysis of coronal brain sections using an specific anti-C3 antibody showing the CA3, CA1, and DG fields of the hippocampus. No induction in the expression of C3 was observed in C/EBPβ knockout mice after KA injection, compared with their wild type littermate controls. *Scale bar*, 50 μm. *Inset scale bar*, 25 μm. **b** RT-PCR analysis of C3 mRNA content in the hippocampus of adult C/EBPβ^+/+^ and C/EBPβ^−/−^ mice treated or not with KA. The graphic shows the mean of three different experiments

In order to determine the causative implication of C/EBPβ in the KA-induced expression of the C3 gene, we analyzed the effect of KA injection in the hippocampal expression of C3 gene in C/EBPβ^−/−^ mice. As can be observed in Fig. 5a, C3 protein levels were clearly increased relative to vehicle-injected controls in the hippocampus 72 h following KA injection of C/EBPβ^+/+^ animals. On the contrary, no increase in C3 was observed in the hippocampus of C/EBPβ^−/−^ mice. These data further support the notion that the transcription factor C/EBPβ is a major regulator of C3 gene expression in the brain [54]. Furthermore, our results show that mice lacking C/EBPβ do not present an increase in C3 mRNA levels after the lesion (Fig. 5b), further indicating that indeed C/EBPβ is essential to induce C3 expression in the hippocampus and also suggesting a local production of C3.

**Fig. 6:**
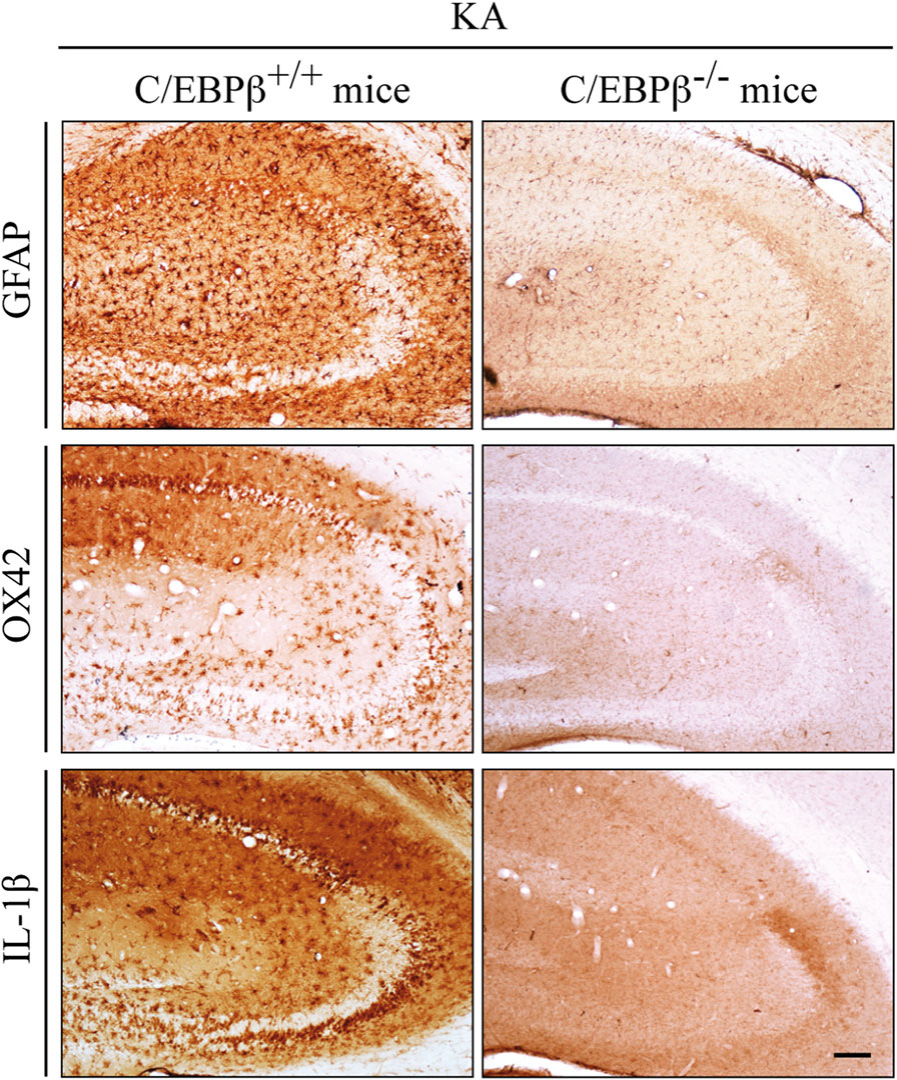
Increased hippocampal inflammation after KA injection in C/EBPβ wild type mice. Mice were injected with KA in the right hemisphere, sacrificed 72 h later, and immunohistochemistry analysis were performed using specific anti-GFAP, anti-OX42, and anti-IL1β antibodies. No induction of inflammation was observed in C/EBPβ knockout mice after KA injection, compared with their wild type littermate controls. *Scale bar*, 100 μm

One of the events that takes place in the hippocampus after KA injury is the activation of microglial cells and the liberation of proinflammatory cytokines, which is in part responsible for the neuronal degeneration. Glial activation (as shown by GFAP- and OX42-positive cells) and induction of IL1β (a very potent proinflammatory agent) were clearly observed in the hippocampus of wild type mice 72 h after KA injection (Fig. 6). This strong neuroinflammation process was completely absent in the hippocampus of C/EBPβ^−/−^ mice.

## Discussion

In this work, we focused on the analysis of coexpression of C/EBPβ and C3 in an in vivo model of excitotoxicity. We chose this model since previous studies from our laboratory demonstrate an important role of C/EBPβ in excitotoxic processes [16]. Our results show that the injection of KA induces the expression of C/EBPβ and C3 genes in the hippocampus of mice and rats. We also show that the induction of both genes occurs in the same cell and is impaired in cells depleted of C/EBPβ. These data, together with a previous study from our laboratory showing a direct regulation of mouse C3 promoter by C/EBPβ in vitro [54], indicate that C3 is a downstream target of C/EBPβ after a brain injury. Because C3 functions as a central complement component, most probably this gene plays an important role in the proinflammatory effects of C/EBPβ in the central nervous system.

The C/EBPβ gene plays an important role in neuroinflammation [61]. Its expression and the activity of the protein are upregulated by brain injury and inflammatory conditions and, additionally, C/EBPβ induces the expression of genes coding for proinflammatory cytokines [16, 62]. The induction by KA of C/EBPβ expression in the hippocampus of mice and rats shown in this work is in agreement with previous data from our laboratory showing, in mice injected with KA, an increase in the nuclear content of C/EBPβ mainly in the granular neurons of the dentate gyrus, but also in astrocytes and microglia cells [16]. Here we have extended these results by showing that C/EBPβ is also clearly induced in the CA1 and CA3 regions of the mouse hippocampus, which is in agreement with the described presence of C/EBPβ transcripts in these subfields of the adult mouse hippocampus, although at lower levels than in the dentate gyrus [63]. In contrast with the mouse, in the rat hippocampus the induction of C/EBPβ expression by KA was observed only in the CA3 subfield of the hippocampus, indicating a clear difference between both species. Interestingly, this enhancement in C/EBPβ protein levels occurs in neurons, as we previously observed in the dentate gyrus of mice. All these data are consistent with previous work showing the induction of C/EBPβ gene expression by KA and proinflammatory agents in primary cultured glial cells [13, 14, 16].

C/EBPβ regulates the expression of various cytokine and chemokine genes, which play an important role in the regulation of innate and adaptive immunity [64–66]. The regulation of many of them is probably directly dependent of C/EBPβ. Such is the case of the proinflammatory cytokines IL-1β, IL6, and TNFalpha, where active C/EBPβ binding sites have been described in the corresponding promoters of the human genes [67, 68]. The results presented in this work showing a parallel induction of C/EBPβ and C3 genes by KA and a lack of induction of C3 in C/EBPβ knockout mice, together with the commented previous results from our laboratory showing that C/EBPβ directly regulates the expression of C3 [54], clearly include C3 gene among those genes directly regulated by C/EBPβ.

Accumulation of C3 protein after KA injection was observed in both glial and neuronal cells as shown by double labeling with a C3-specific antibody and cell type-specific markers. In the rat CA3 subfield, labeling with neurotrace, a specific neuronal marker, together with a C3 antibody, clearly indicates the presence of C3 protein inside the neurons and also in the surrounding area. Given the massive neuronal cell death that takes place in this region of the hippocampus, these observations suggest that C3 may be localized in those neurons that are already degenerating. In the case of glial cells, C3 accumulates in activated astrocytes and microglial cells. When double immunostaining for C3 and C/EBPβ was performed in the CA3 region of the mouse hippocampus after KA injection, both proteins were found in the same cells (C3 in the cytosol and C/EBPβ in the nucleus). These results further suggest that C3 expression could be regulated by C/EBPβ both in glial and neuronal cells and are consistent with other results showing that in the brain C3 can be produced by neuronal and glial cells [23, 69]. Specifically, in situ hybridization studies [32, 70–72] have shown that the C3 component is locally produced in glial cells and neurons and that the presence of C3 is not merely due to leakage of plasma proteins because of blood brain barrier damage.

Regarding the regulation of C3 gene expression by C/EBPβ, our results are in agreement with previous work showing that C/EBPβ plays an important role in the regulation of C3 expression by IL-1β [73, 74] and also with data from microarray analysis by our laboratory showing that the overexpression of C/EBPβ in neuroblastoma cells and its absence in the mouse hippocampus correlates, respectively, with an increase and a decrease in the amount of C3 transcripts [15, 54]. Moreover, the results presented here show that the increase in C3 mRNA after KA injection observed in control mice is not detected in C/EBPβ-deficient mice, further suggesting that C/EBPβ is essential to induce C3 expression in the hippocampus after KA injection.

Consistent with the hypothesis that the C3 gene could play an important role as mediator of the proinflammatory effect of C/EBPβ, some data in the literature implicate C3 as an active factor in neuronal damage in experimental models of inflammation [75, 76] and traumatic brain injury [77, 78]. In this regard, it has been shown that C3-deficient mice show less neuronal loss and microglial activation in several models of brain injury in different brain areas, including the hippocampus [75, 79–81]. However, the function of the C3 is complex and a protective effect of C3 has been described in experimental models of Alzheimer’s disease, probably as a consequence of favoring the clearance of Aβ plaque [82, 83].

Excitotoxicity has been implicated as a pathogenic mechanism associated with different brain disorders, including acute brain injury and neurodegenerative diseases, including epilepsy [84, 85]. Glutamate is the major excitatory neurotransmitter in the central nervous system and a primary driver of the excitotoxic process. Although glutamate plays a central role in excitatory neurotransmission, alterations in glutamate homeostasis can have significant repercussions on neurons through the generation of neurotoxic and excitotoxic cascades. Particularly sensitive to KA injection are the hippocampal CA1 and CA3 regions, and the hilar neurons of the dentate gyrus [86]. Glial cells have an important role in the course of KA-induced hippocampal neurodegeneration. Activated astrocytes and microglial cells proliferate and increase the expression of genes involved in this degeneration process.

Regarding epilepsy, our results are in accordance with previous works showing an implication of the complement system in epilepsy. Specifically, an increase in C3 expression in the brain of patients with temporal lobe epilepsy [87, 88] has been shown, which is the common type in adults and is characterized by neuronal loss and gliosis in the hippocampus. The current literature demonstrates that injury to the brain results in a temporally orchestrated genetic response by neurons and glial cells, which involves signaling pathways that promote cell death and survival, yet a more complete elucidation of the exact molecular mechanisms is required. In particular, determination of the transcription factors involved and the downstream gene effectors they induce will provide invaluable insight into the neuroprotective signaling pathways. This will have immense public health implications, prevent ill health, and reduce the cost to society. In this regard, our results suggest that C/EBPβ and C3 might be important molecular new targets responsible for the neurodegeneration that occurs after a brain injury. It is important to note that this work shows that C/EBPβ regulates in vivo the expression of C3 in the hippocampus after an excitotoxic injury since C/EBPβ knockout mice do not show the induction of C3 after KA injection.

## Conclusions

Collectively, our findings further demonstrate an activation of the C3 gene in vivo by C/EBPβ and suggest a role for C3 as a mediator of this transcription factor following a brain insult.

## Additional file

Additional file 1: Double-immunofluorescence analysis showing C3 expression near microglia and blood vessels 72 h after KA injection in adult rats. All images represent the maximum intensity projection, and orthogonal views are also shown, generated by projecting *z*-series in the *x*- and *y*-planes. Scale bar, 5 μm. (TIF 2327 kb)

## Abbreviations

C/EBPβ: CCAAT/enhancer-binding protein β
C3: Complement component 3
KA: Kainic acid
