# Supplementary figures and images for: Complement component 3 (C3) expression in the hippocampus after excitotoxic injury: role of C/EBPβ
Source: J Neuroinflammation. 2016 Oct 21;13:276. doi: 10.1186/s12974-016-0742-0 (PMC5073972; doi:10.1186/s12974-016-0742-0)

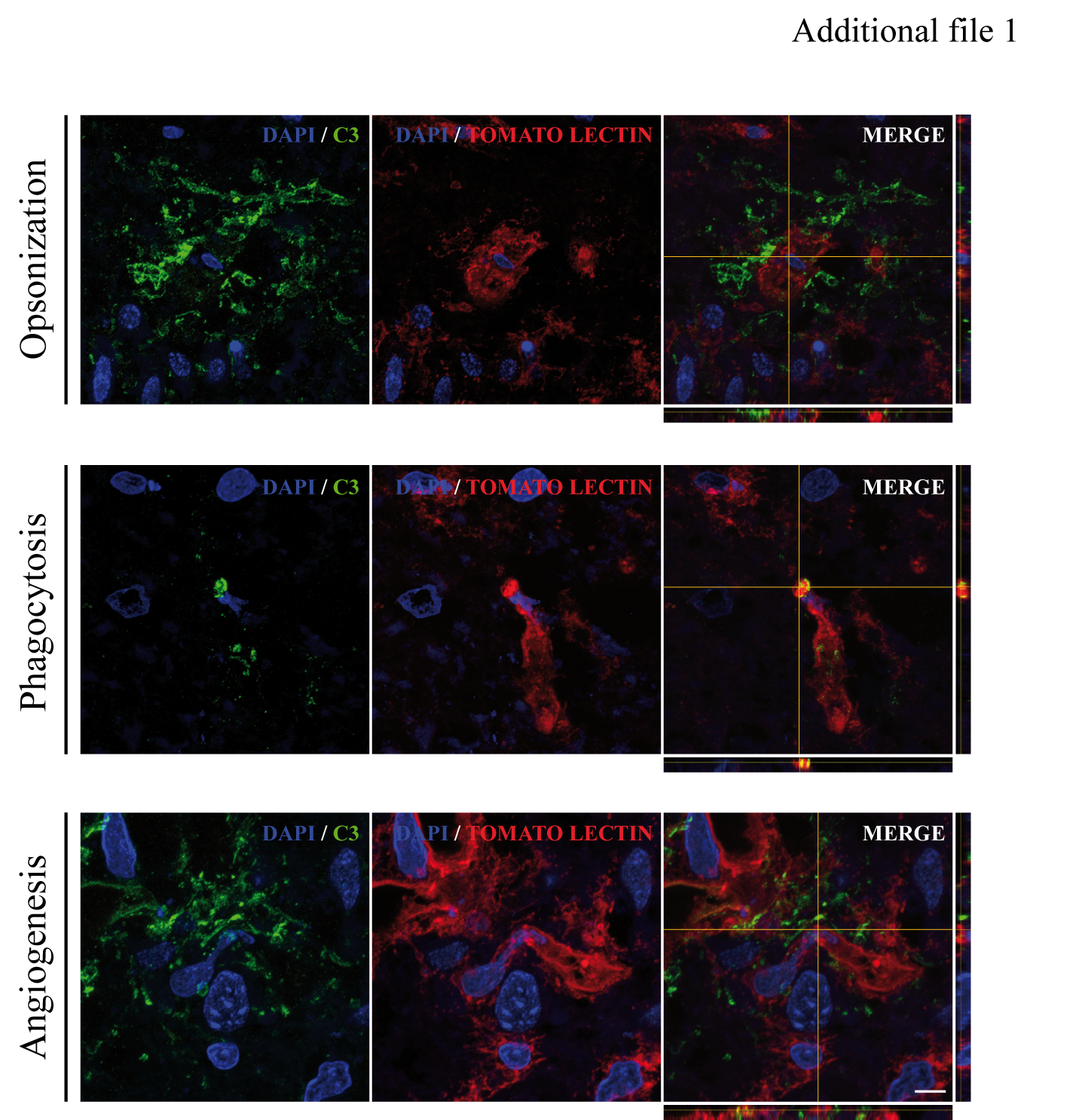

Supplement: Additional file 1: — Double-immunofluorescence analysis showing C3 expression near microglia and blood vessels 72 h after KA injection in adult rats. All images represent the maximum intensity projection, and orthogonal views are also shown, generated by projecting z-series in the x- and y-planes. Scale bar, 5 μm. (TIF 2327 kb) [file 12974_2016_742_MOESM1_ESM.tif]
